# Occult Femoral Neck Fracture Misdiagnosed as Septic Arthritis: A Case Highlighting Diagnostic Challenges in Busy Emergency Settings

**DOI:** 10.7759/cureus.92335

**Published:** 2025-09-15

**Authors:** Abdul Rehman, Muhammad Amjad, Muzamil Aslam Chaudhary, Fazeelah Bibi

**Affiliations:** 1 Emergency Department, St. Luke's General Hospital, Kilkenny, IRL; 2 Emergency Department, Wrightington, Wigan and Leigh NHS Foundation Trust, Wigan, GBR; 3 Emergency Department, Pakistan Institute of Medical Sciences Hospital, Islamabad, PAK

**Keywords:** ct pelvis, emergency medicine, hip trauma, missed fracture, occult femoral neck fracture, septic arthritis

## Abstract

Occult femoral neck fractures are a diagnostic challenge in busy emergency settings, especially when initial imaging is unremarkable. Confounding clinical features, temporary response to analgesia, and raised inflammatory markers can lead to misdiagnosis, delaying definitive care. We present a case of a 52-year-old male who initially presented with right hip pain following a fall. His radiographs were unremarkable, and he was discharged after pain control. He re-presented three days later with worsening hip pain, inability to weight bear, and confusion. Raised inflammatory markers led to a presumptive diagnosis of septic arthritis, but a computed tomography (CT) scan ultimately revealed a basicervical femoral neck fracture. The patient underwent successful open reduction and internal fixation (ORIF). This case underscores the importance of maintaining a high index of suspicion for occult fractures, especially in patients with persistent or worsening pain and early representation. It also highlights the limitations of plain radiography in hip trauma. CT or magnetic resonance imaging (MRI) should be considered when clinical signs are incongruent with imaging findings.

## Introduction

Femoral neck fractures are a common injury in adults, accounting for a significant proportion of emergency orthopedic presentations. These injuries often present after a low-energy trauma and require prompt diagnosis to avoid complications such as avascular necrosis and impaired mobility [1]. While most fractures are evident on plain radiographs, up to 10% may be occult [2]. In these cases, computed tomography (CT) or magnetic resonance imaging (MRI) is essential for accurate diagnosis [3,4]. Occult fractures are particularly challenging in high-volume emergency departments, where subtle signs may be missed and overshadowed by normal imaging and initial relief from analgesics.

Misdiagnosis can occur, especially in patients presenting with systemic signs, such as raised inflammatory markers or altered mental status, which may mimic infectious conditions like septic arthritis [5]. Early and accurate diagnosis is essential to prevent complications and optimize outcomes. We present a case of an occult femoral neck fracture initially misdiagnosed as septic arthritis, highlighting key diagnostic pitfalls and important learning points for emergency physicians. This case underscores the need for diagnostic vigilance in patients with persistent hip pain and normal initial imaging.

## Case presentation

A 52-year-old previously healthy male presented to the emergency department with right hip pain following a mechanical fall at home two days ago. He had slipped on a wet floor and landed on his right side. He denied loss of consciousness, head trauma, or chest or neck pain. His past medical history was unremarkable, and he was fully ambulatory prior to the incident and had no mobility limitations.

On examination, he was hemodynamically stable, alert, and oriented. There was tenderness over the right hip joint with a painful and mildly restricted range of motion, but no bruising or deformity was noticed. He was able to do a straight leg raise in the right hip and was able to partially weight-bear with crutches. Neurological and vascular examinations of the lower limb were normal.

**Figure 1:**
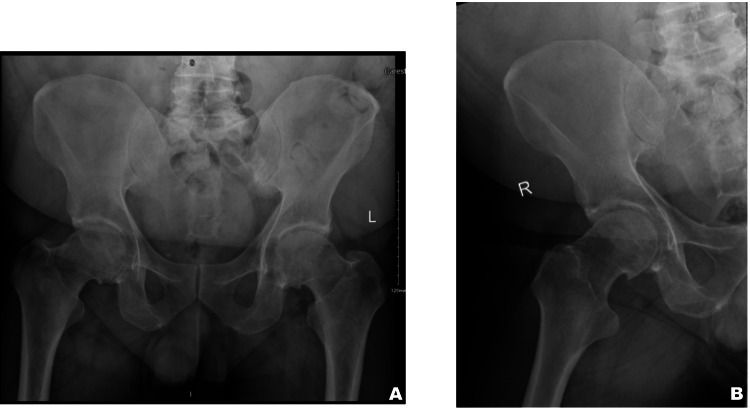
Pelvic and right hip radiographs showing no evidence of acute fracture.

We performed pelvic and right hip radiographs, reviewed by the radiologist and reported as normal, with no evidence of acute fracture or dislocation (Figure 1). The patient was administered oral and parenteral analgesia, which led to significant symptomatic relief. On reassessment after analgesia, his range of motion in the right hip improved, and he was able to walk using one crutch. He was discharged home with analgesics and mobility aids, with advice for physiotherapy and outpatient follow-up if symptoms persisted.

Three days later, the patient re-presented to the ED with worsening right hip pain despite regular analgesia, inability to weight bear, and new-onset confusion. He was afebrile, with a heart rate of 104 beats per minute, normal blood pressure, and oxygen saturation. Cognitive assessment showed mild cognitive impairment, without any focal neurological signs. Examination of the right hip revealed severe pain on passive and active movement in all directions, with marked guarding.

Laboratory investigations showed elevated C-reactive protein (CRP) of 175 mg/L, with normal white cell count, renal function, and liver function tests (Table 1). Chest radiograph and CT brain were also obtained to rule out infection, stroke, or a metabolic cause of confusion. Given the recent normal radiographs, elevated CRP, and a painful joint, septic arthritis of the right hip was initially suspected, and the orthopedic team was consulted for joint aspiration.

**Table 1:** Laboratory investigations MCV: mean corpuscular volume; CRP: C-reactive protein; AST: aspartate transaminase; ALT: alanine transaminase; Na: sodium; K: potassium.

Test	Result	Normal range
WBC count	8.5 × 10^9^/L	4-10 × 10^9^/L
Hemoglobin	14.0 g/dl	13-17 g/dl
Platelets	330 × 10^9^/L	150-450 × 10^9^/L
MCV	92 fl	83-101 fl
CRP	175 mg/L	<5 mg/L
Serum Na	136 mmol/L	135-145 mmol/L
Serum K	4.1 mmol/L	3.5-5.0 mmol/L
Chloride	103 mmol/L	98-107 mmol/L
Bicarbonate	24 mmol/L	22-28 mmol/L
Urea	5.6 mmol/L	2.5-7.8 mmol/L
Creatinine	65 mmol/L	45-105 umol/L
AST	44 U/L	15-50 U/L
ALT	43 U/L	5-55 U/L

**Figure 2:**
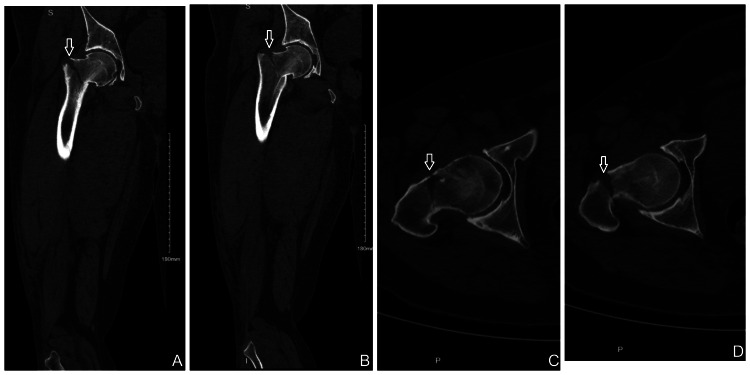
Coronal (A) and (B) and axial (C) and (D) computed tomography images of the right hip demonstrating a basicervical fracture of the femoral neck (white arrows).

**Figure 3:**
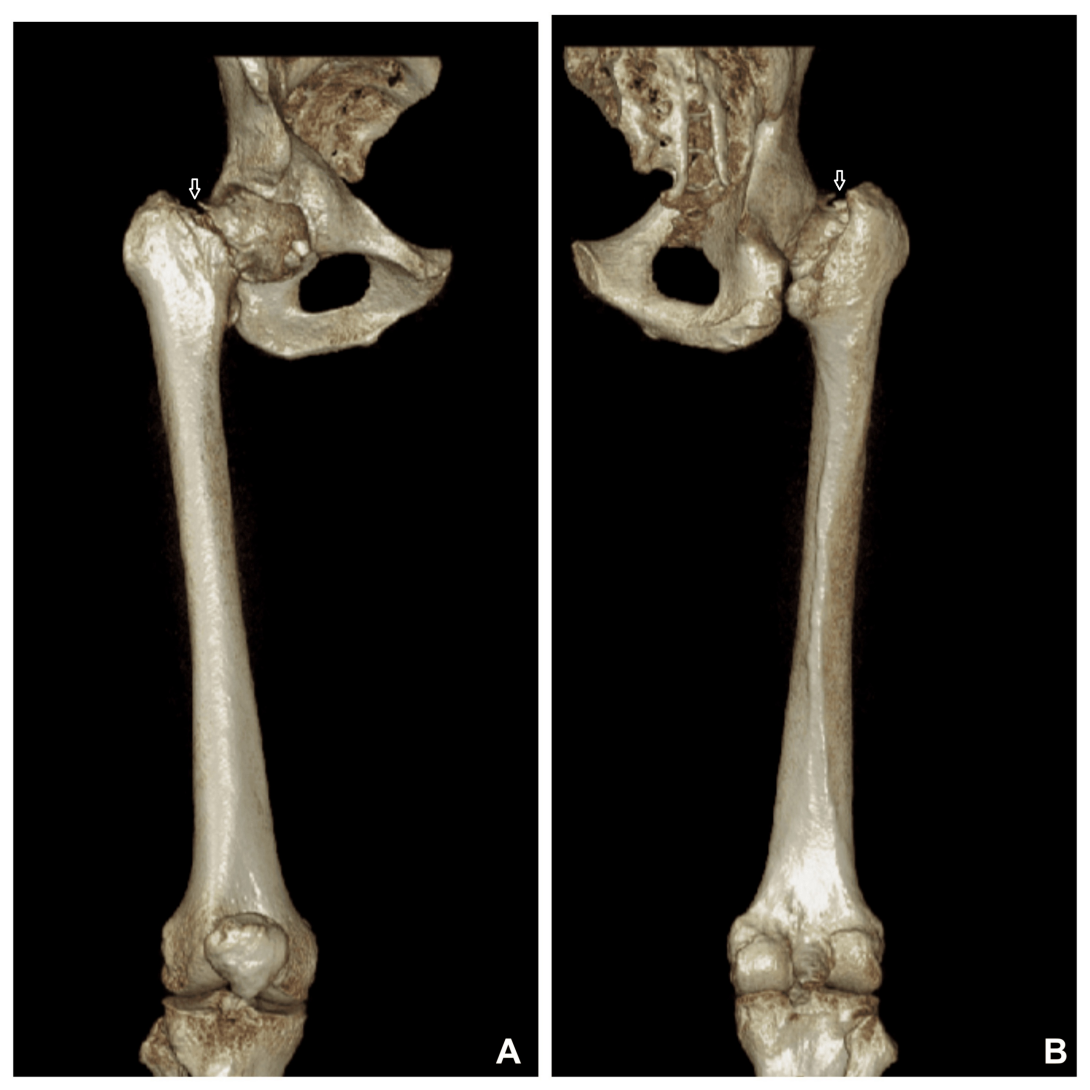
CT scan (3D bone window) showing basicervical fracture (white arrows) of the right femoral neck.

A CT scan was performed prior to arthrocentesis, revealing a basicervical femoral neck fracture not seen on initial X-rays (Figures 2, 3).

**Figure 4:**
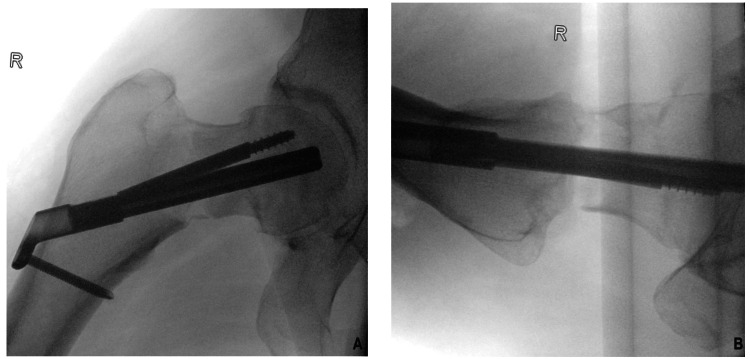
Immediate post-operative fluoroscopic images showing internal fixation, using DHS, of the right hip. DHS: dynamic hip screw.

Before proceeding with surgery on the right hip, arthrocentesis was performed to rule out septic arthritis. Synovial fluid analysis showed no evidence of septic arthritis (Table 2). The following day, he underwent closed reduction and internal fixation of the right hip with a dynamic hip screw (DHS) (Figure 4). Post-operative recovery was uneventful. His confusion resolved after adequate pain control and mobilization. The patient was discharged to a rehabilitation unit for physiotherapy and made a full recovery over the subsequent weeks. Upon the latest follow-up visit at four months post-operatively, he reported a return to his routine activities and absence of any pain in his right hip.

**Table 2:** Synovial fluid findings. PMNs: polymorphonuclear leukocytes.

Synovial fluid test	Result	Normal
Color	Yellow	Clear
Clarity	Opaque	Transparent
Viscosity	Low	High
White blood cells	45 × 10^9^/L	<2 × 10^9^/L
Percentage of PMNs	<50%	<25%
Culture result	Negative	Negative

## Discussion

Our case illustrates the diagnostic challenges of occult femoral neck fractures, particularly in patients who initially present with mild-moderate pain and respond to analgesia. Fractured neck of femur (NOF) is associated with significant morbidity and mortality. Occult hip fractures are defined as fractures not visible on plain radiographs but detectable by advanced imaging modalities such as computed tomography (CT) or magnetic resonance imaging (MRI). These account for up to 2-10% of all femoral neck fractures [2]. Therefore, in cases where clinical suspicion remains high despite negative initial radiographs, further imaging is warranted.

Magnetic resonance imaging (MRI) is generally regarded as the preferred modality in such situations, with some reports describing sensitivity and specificity approaching 100% [3]. Nonetheless, these studies are limited by small sample sizes and methodological issues such as selection and verification bias. Computed tomography (CT) is another option, as it can reveal fractures not obvious on plain radiographic films. Its limitations include possible missed injuries in patients with marked osteoporosis, due to reduced resolution of the trabecular structure, or if the fracture lies in the same plane as the CT slices. Despite being more readily available in many emergency departments compared to MRI, the supporting evidence for CT is sparse and underpowered.

National Institute for Health and Care Excellence (NICE) and the British Orthopaedic Association Standards for Trauma advise offering MRI to patients with negative X-rays when clinical suspicion persists. CT is suggested as a suitable alternative if MRI is contraindicated or cannot be performed within 24 hours. NICE further emphasizes the need for additional research to assess whether CT could represent a clinically and cost-effective substitute for MRI [4].

Our patient's initial improvement in pain following analgesia contributed to diagnostic anchoring and premature discharge. However, pain relief can mask the severity of an underlying injury. Post-analgesia physical examination and normal radiographs may provide false reassurance, particularly if the clinician does not repeat functional assessment.

Elevated CRP and new-onset confusion on second presentation led to a presumptive diagnosis of septic arthritis [6]. Septic arthritis after femoral neck fracture is a very rare complication, and only a handful of cases have been reported. Colak et al. reported three cases, and Hearth et al. reported two cases. The cases in both reports were all immunocompromised, and the ages varied from 48 to 96 years old [7, 8]. While appropriate in the differential, it is important to remember that trauma and fractures can elevate inflammatory markers in the absence of infection [9-11]. Additionally, confusion in elderly or pain-distressed patients may be multifactorial and should not exclude orthopedic pathology.

Delayed diagnosis of femoral neck fractures may lead to displacement, avascular necrosis, or nonunion, increasing morbidity. Early identification allows for timely surgical intervention, reducing hospital stay and improving functional outcomes [12,13]. Clinicians should maintain a low threshold for advanced imaging in patients with persistent pain after trauma, even when radiographs are normal.

## Conclusions

Occult femoral neck fractures should be considered in all patients with persistent hip pain after trauma, even if initial radiographs appear normal. CT or MRI should be promptly utilized in cases with incongruent clinical findings. Clinicians should remain cautious about interpreting symptom relief following analgesia and avoid anchoring to alternative diagnoses like septic arthritis solely based on inflammatory markers or new onset of confusion. A multidisciplinary approach and early imaging can prevent missed diagnoses and ensure timely, appropriate care.

This case highlights the importance of clinical vigilance in assessing patients with hip trauma. It also underscores the importance of advanced imaging in patients with persistent hip pain and inconclusive radiographs. Normal radiographs should not preclude further investigation in patients with persistent pain, inability to weight bear, or atypical symptoms. Early CT or MRI is crucial to diagnose occult fractures, improve outcomes, and prevent misdiagnosis, such as septic arthritis, especially in patients with nonspecific signs like confusion or elevated inflammatory markers.
